# Rapid fabrication of MgO@g-C_3_N_4_ heterojunctions for photocatalytic nitric oxide removal

**DOI:** 10.3762/bjnano.13.96

**Published:** 2022-10-18

**Authors:** Minh-Thuan Pham, Duyen P H Tran, Xuan-Thanh Bui, Sheng-Jie You

**Affiliations:** 1 Department of Civil Engineering, Chung Yuan Christian University, Taoyuan 32023, Taiwan; 2 Department of Environmental Engineering, Chung Yuan Christian University, Taoyuan 32023, Taiwan; 3 Center for Environmental Risk Management, Chung Yuan Christian University, Taoyuan 32023, Taiwan; 4 Faculty of Environment and Natural Resources, Ho Chi Minh City University of Technology (HCMUT), VNU-HCM, 268 Ly Thuong Kiet Street, District 10, Ho Chi Minh City 700000, Viet Nam

**Keywords:** g-C_3_N_4_, MgO, nitric oxide, photocatalyst, visible light

## Abstract

Nitric oxide (NO) is an air pollutant impacting the environment, human health, and other biotas. Among the technologies to treat NO pollution, photocatalytic oxidation under visible light is considered an effective means. This study describes photocatalytic oxidation to degrade NO under visible light with the support of a photocatalyst. MgO@g-C_3_N_4_ heterojunction photocatalysts were synthesized by one-step pyrolysis of MgO and urea at 550 °C for two hours. The photocatalytic NO removal efficiency of the MgO@g-C_3_N_4_ heterojunctions was significantly improved and reached a maximum value of 75.4% under visible light irradiation. Differential reflectance spectroscopy (DRS) was used to determine the optical properties and bandgap energies of the material. The bandgap of the material decreases with increasing amounts of MgO. The photoluminescence spectra indicate that the recombination of electron–hole pairs is hindered by doping MgO onto g-C_3_N_4_. Also, NO conversion, DeNOx index, apparent quantum efficiency, trapping tests, and electron spin resonance measurements were carried out to understand the photocatalytic mechanism of the materials. The high reusability of the MgO@g-C_3_N_4_ heterojunction was shown by a five-cycle recycling test. This study provides a simple way to synthesize photocatalytic heterojunction materials with high reusability and the potential of heterojunction photocatalysts in the field of environmental remediation.

## Introduction

The rapid development of industrialization has been continuously increasing the combustion of fossil fuels, which leads to a large extent of nitrogen oxide emissions. This particular type of air pollutant leads to environmental damage (e.g., smog and acid) and health problems (e.g., COPD and cardiovascular diseases) [1–3]. Presently, there are different approaches to mitigate NO pollution, including catalyst/non-catalyst [4], oxidation [5], bioprocesses [6], adsorption [7], absorption [8], and non-thermal plasma technologies [9]. Photocatalytic oxidation is considered a promising approach due to its ability to degrade various air pollutants with light under ambient conditions [10].

Due to its unique properties, such as high chemical stability and low synthesis cost, graphitic carbon nitride has attracted considerable attention in the realm of environmental remediation [11–13]. It is an organic semiconductor that effectively absorbs visible light due to its small bandgap below 2.7 eV. Because of this, it has been consistently regarded as a catalyst with excellent optical properties [14–15]. Unfortunately, its narrow bandgap leads to rapid recombination of electron–hole (e^−^–h^+^) pairs, and the valence band potential of g-C_3_N_4_ (+1.75 eV) is more negative than that of H_2_O/•OH (+2.40 eV), reducing the photocatalytic efficiency [16–17]. A well-known approach for overcoming this problem in order to achieve increased photocatalytic performance is to couple two semiconductors with optimal band alignment.

MgO is an alkaline metal oxide with wide bandgap (3.5–5 eV), high availability, non-toxicity, low cost, and native structural defects [18–19]. The large bandgap energy is the limitation of MgO, reducing the photocatalytic performance and applicability of MgO [20]. Various efforts have been made to enhance the absorption in the visible light region, including nonmetal and noble-metal doping, metal deposition, and formation of heterojunctions [21–22]. The construction of heterojunction structures has shown its effectiveness in improving photocatalytic performance by enhancing the separation of charge carriers and optimizing the redox potential by coupling two or more semiconductors [23–24], such as Bi_2_MoO_6_-based [25–29], BiOCl-based [30–31], g-C_3_N_4_-based [32–34], ZnO-based [35–37], TiO_2_-based [38–39], and MgO-based heterostructured photocatalysts [40]. Among these, the combination of MgO and g-C_3_N_4_ with a lower bandgap is an efficient process for improving the photocatalytic performance. Li and co-workers reported an improvement in the photocatalytic efficiency of MgO@g-C_3_N_4_ for the photoreduction of CO_2_ under visible light [33]. Similarly, MgO-modified g-C_3_N_4_ nanostructures enhanced the removal efficiency for NO*_x_*, NO, and NO_2_ [12]. However, these studies only focused on the synthesis of MgO from Mg(NO_3_)_2_·6H_2_O, increasing time and cost of the synthesis process. Commercial MgO as a precursor material for MgO@g-C_3_N_4_ heterojunctions has not been studied. Furthermore, there are no relevant reports on the fabrication of the MgO@g-C_3_N_4_ heterojunction via one-step pyrolysis nor on the photocatalytic pathway of the MgO@g-C_3_N_4_ heterojunction for photocatalytic NO removal under visible light.

In this study, a MgO@g-C_3_N_4_ heterojunction was synthesized via a one-step pyrolysis method using commercial MgO and urea and, subsequently, characterized. Charge transfer dynamics in the heterojunction and band structure were investigated to understand the effect of the heterojunction on the photocatalytic activity. Finally, the photocatalytic pathway of the MgO@g-C_3_N_4_ heterojunction was studied via trapping test, electron spin resonance (ESR) measurements, and other methods. This work might be helpful for the development of MgO@g-C_3_N_4_ heterojunction materials.

## Experimental

### Synthesis of MgO/g-C_3_N_4_

For the synthesis of g-C_3_N_4_, 30 g of urea was ground manually for 30 min and then placed in a 100 mL crucible. Then, the sample was annealed at 550 °C for two hours and let to cool to room temperature.

**Figure 1:**
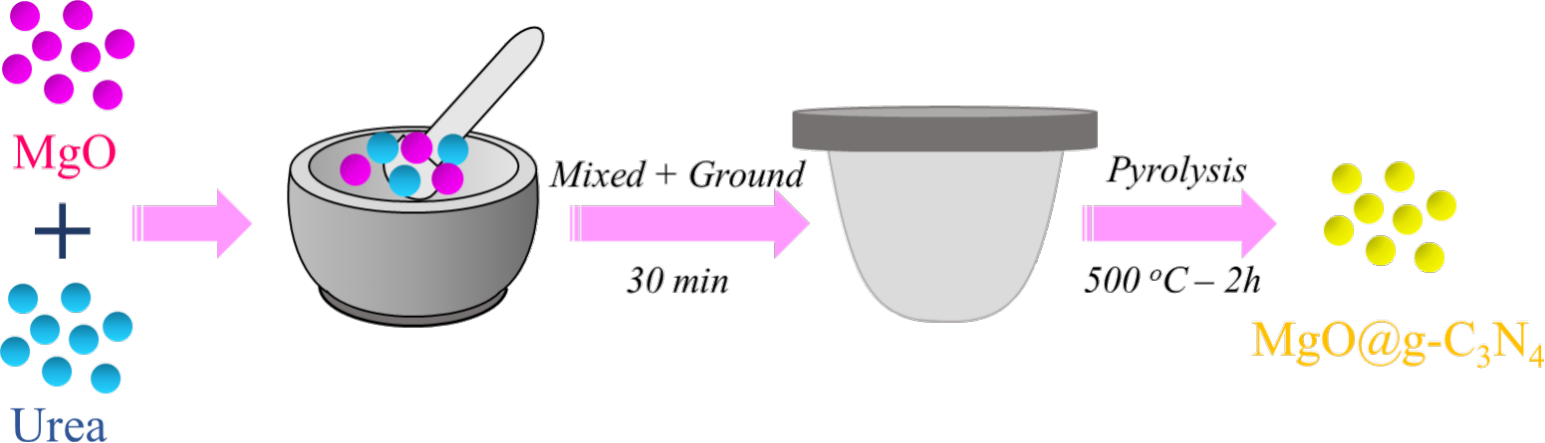
Schematic diagram of pyrolysis synthesis process for MgO@g-C_3_N_4_ heterojunctions.

For preparation of the MgO@g-C_3_N_4_ heterojunction material, both MgO and urea were mixed, ground, and placed in a 100 mL crucible, followed by annealing at 550 °C for two hours (Figure 1). Then, the samples were let to cool to room temperature naturally. Different mass ratios of MgO and g-C_3_N_4_ were prepared, that is, 1%, 3% and 5%, named as *x*-MgO@g-C_3_N_4_ (*x* = 1, 3, and 5%).

### Characterization

A variety of analytical techniques have been employed to evaluate the morphology and the physical, chemical, and optical properties of the materials. Scanning electron microscopy (SEM) and high-resolution transmission electron microscopy (HR-TEM) were used to assess the morphology of the materials. The crystal phase of the materials was determined by X-ray diffraction (XRD) with a measurement range of 10°–80°. Fourier-transform infrared spectroscopy (FTIR) was used to determine the chemical bond composition of the materials. Differential reflectance spectroscopy (DRS) determined the change in the bandgap of the materials. The elements of the materials were identified by high-resolution X-ray photoelectron spectroscopy (HR-XPS). The photoluminescence (PL) spectra of the materials was carried out in the form of fluorescence analysis with an excitation wavelength range of 200–900 nm. Finally, the photocatalytic mechanism was determined by trapping tests and ESR measurements.

#### Photocatalytic performance

The photocatalytic activity of as-prepared MgO@g-C_3_N_4_ was evaluated by monitoring NO degradation. The photocatalytic NO removal experiments were performed using a 4.5 L reaction chamber and a Xenon lamp (300 W) as the visible light source. The initial NO concentration was 500 ppb, the flow rate was 1.5 L·min^−1^, and the dosage of catalysts was 0.2 g for all experiments. Before each catalytic experiment, 0.2 g of the sample was dispersed in 10 mL of DI water, evaporated at 80 °C, and placed in the dark to achieve adsorption–desorption equilibrium. Finally, the sample was illuminated by a Xenon lamp (300 W) for 30 min.

Trapping experiments were performed to evaluate the photocatalytic process mechanism for NO degradation. Three trapping agents were used representing different active species, namely isopropyl alcohol (IPA) for the hydroxyl radical (•OH), potassium dichromate (K_2_Cr_2_O_7_) for electrons (e^−^), and potassium iodide (KI) for holes (h^+^). The photocatalytic NO degradation experiments were performed under the previously described conditions.

The photocatalytic NO degradation efficiency (η), the yield of NO_2_ conversion (γ), the apparent quantum efficiency (AQE, φ), and the DeNOx index (αDeNOX αDeNOx) were calculated by using Equations 1–4: [41–43]:


[1] $$\eta =\frac{C_{\text{NO, i}}-C_{\text{NO, f}}}{C_{\text{NO, i}}}\cdot 100\%,$$

[2] $$\gamma =\frac{C_{{\text{NO}}_{2}\text{, f}}-C_{{\text{NO}}_{2}\text{, i}}}{C_{\text{NO, i}}-C_{\text{NO, f}}}\cdot 100\%,$$

[3] $$\varphi_{\text{app}}=\frac{N_{\text{A}}\int_{0}^{t}\left(C_{\text{NO, i}}-C_{\text{NO, f}}\right)V_{t}}{\text{photon flux}\cdot \text{irradiation area}\cdot t\cdot 1000\text{ M}}\cdot 100\%,$$

[4] $$\alpha_{\text{DeNOx}}=\eta \left(1-3\gamma \right)C_{\text{NO, i}},$$

where *C*_NO_ is the concentration of NO (ppb), *C*_NO2_ is the concentration of NO_2_ (ppb), the index “i” represents the initial concentration, and the index “f” represents the final concentration. *N*_A_ is the Avogadro constant (mol^−1^), *V_t_* is the flow rate of NO (L·min^−1^), and *M* is the molecular weight of NO (g·mol^−1^). The photon flux in the photocatalytic experiment is 2.72·10^19^ cm^−2^·min^−1^, the irradiation area for the 12 cm diameter petri dish is 113.1 cm^2^.

In addition, the bandgap energy of materials was calculated by using the Tauc and the Kubelka–Munk equation as described in Equations 5–7 [43]:


[5] $$E=h\nu =\frac{hc}{\lambda },$$

[6] $${\left(\alpha h\nu \right)}^{r}=B\left(h\nu -E_{\text{g}}\right),$$

[7] $${\left(F\left(R\right)h\nu \right)}^{r}=B\left(h\nu -E_{\text{g}}\right),$$

where E is the photon energy (eV), *h* is Planck’s constant (4.132·10^−15^ eV·s), ν is the photon frequency (s^−1^), *c* is the velocity of light (nm·s^−1^), λ is the wavelength (nm), α is the absorption coefficient, *B* is a constant, and *E*_g_ is the bandgap energy (eV), *R* is the reflectance value.

## Results and Discussion

### Photocatalytic performance

**Figure 2:**
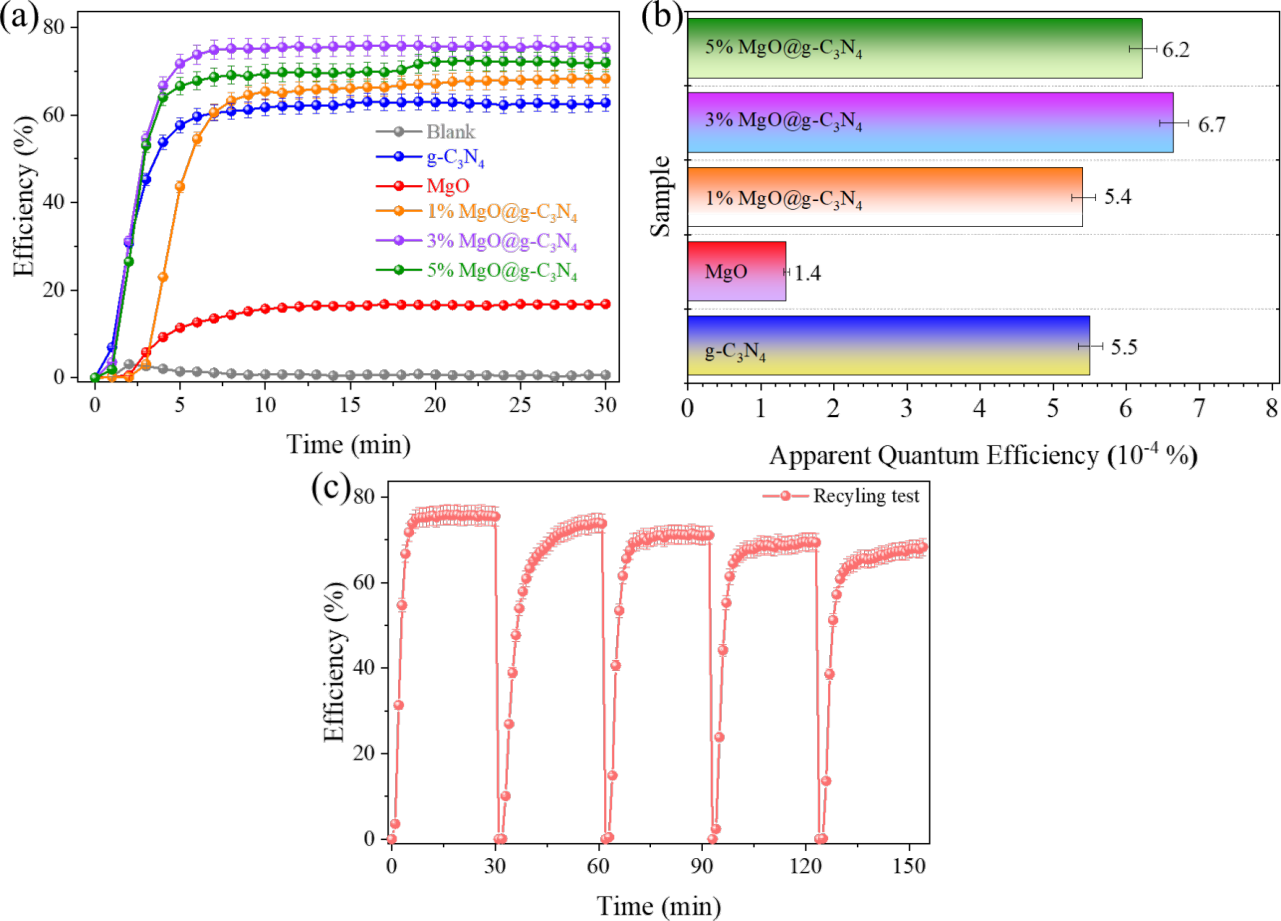
(a) Photocatalytic NO degradation efficiency, (b) apparent quantum efficiency of the materials, and (c) photocatalytic recycling test of 3% MgO@g-C_3_N_4_.

The photocatalytic NO removal efficiency of the materials is shown in Figure 2a. The efficiency gradually increased during the first 5 min of the photocatalytic reaction and remains stable until the end of the photocatalytic reaction. The photocatalytic NO removal efficiency values are 0.6%, 62.8%, 16.8%, 68.4%, 75.4%, and 72.1% for the blank sample, g-C_3_N_4_, MgO, 1% MgO@g-C_3_N_4_, 3% MgO@g-C_3_N_4_, and 5% MgO@g-C_3_N_4_, respectively. The photocatalytic NO removal efficiency is increased by combining MgO with g-C_3_N_4_. The results indicate that MgO@g-C_3_N_4_ heterojunction structures have been successfully synthesized with high photocatalytic NO degradation efficiency under visible light by one-step pyrolysis (see Table 1 for a comparison of the photocatalytic NO removal efficiency values). Also, the AQE has been calculated according to Equation 3. The AQE values (10^−4^%) of g-C_3_N_4_, MgO, 1% MgO@g-C_3_N_4_, 3% MgO@g-C_3_N_4_, and 5% MgO@g-C_3_N_4_ are 5.5, 1.4, 5.4, 6.7, and 6.2, respectively. The AQE results show (Figure 2b) that photons are most efficient in 3% MgO@g-C_3_N_4_. The heterojunction structure has enhanced the photocatalytic activities of the materials [44]. In addition, the photocatalytic reusability of 3% MgO@g-C_3_N_4_ was shown by a five-cycle recycling test under identical experimental conditions (Figure 2c). The photocatalytic NO degradation efficiency in the recycling test is 75.4%, 73.8%, 71.1%, 69.4%, and 68.3% after five cycles, respectively. The photocatalytic NO degradation efficiency decreased by 7% after five cycles. The FTIR spectra and the XRD patterns of the 3% MgO@g-C_3_N_4_ before and after the recycling test are shown in Figure S1a and Figure S1b of Supporting Information File 1, respectively. The results indicate the high reusability of 3% MgO@g-C_3_N_4_ [45].

**Table 1:** Comparison of photocatalytic NO removal of current photocatalyst systems under visible light.

Material	Initial NO concentration (ppb)	Light source	Irradiation time (min)	Dosage (g)	Flowrate (L·min^−1^)	NO removal efficiency (%)	NO_2_ generation	Ref.

BiOIO_3_/ g-C_3_N_4_	600	xenon 300 W	30	0.1	1.2	57	80 (ppb)	[72]
SnO_2_/ g-C_3_N_4_	600	tungsten halogen 150 W	30	0.4	N/A	32	6%	[73]
Ti_3_C_2_@TiO_2_/ g-C_3_N_4_	430	xenon- 300 W	30	N/A	N/A	29	18.7 (ppb)	[74]
rGO/ Fe-doped g-C_3_N_4_	1000	metal halide 250 W	30	N/A	N/A	93.4	N/A	[75]
FAPbBr_3_/g-C_3_N_4_	600	xenon	60	0.1	1.2	58	0.3 (ppb)	[76]
g-C_3_N_4_/SnO_2_	500	xenon- 300 W	30	0.2	0.6	35	2%	[77]
TiO_2_@g-C_3_N_4_	500	xenon- 300 W	30	0.2	0.5	90.2	5.3%	[44]
g-C_3_N_4_@BiOCl/ Bi_12_O_17_Cl_2_	500	tungsten halogen 100 W	30	0.2	1	46.8	N/A	[78]
MoS_2_/g-C_3_N_4_	600	tungsten halogen 150 W	30	0.2	N/A	51.7	N/A	[79]

**Figure 3:**
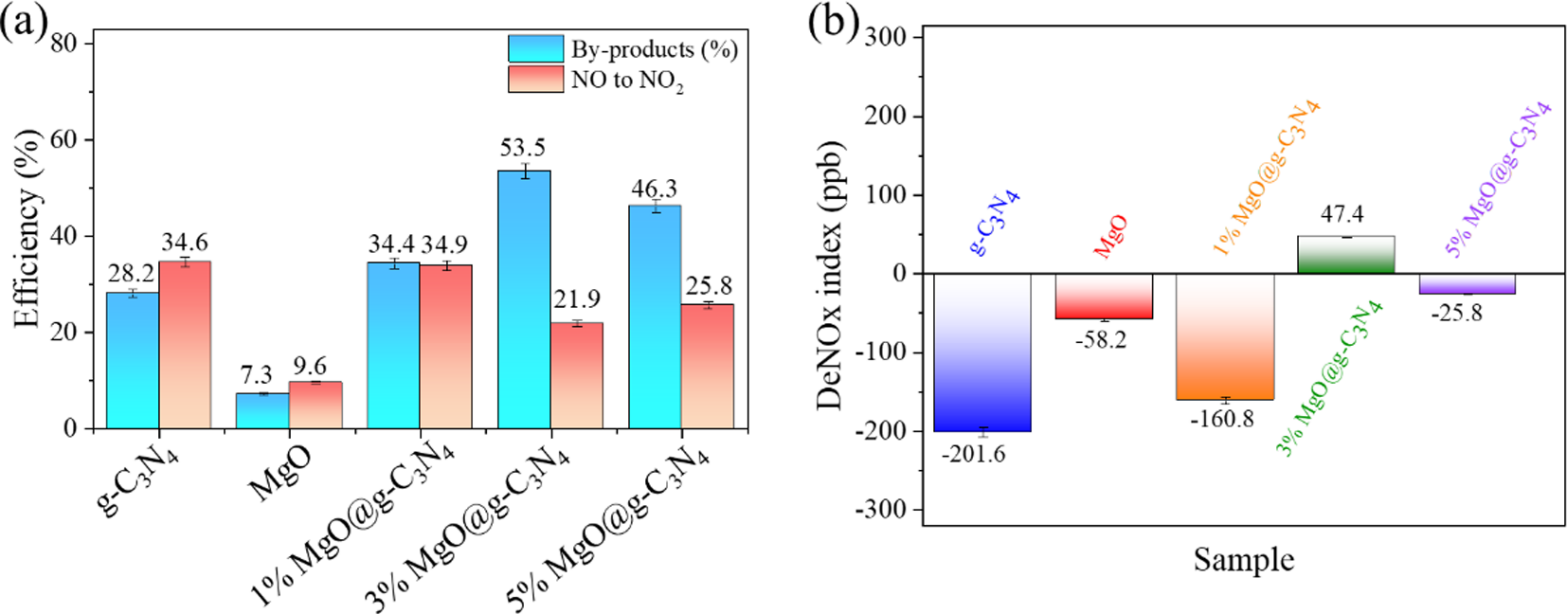
(a) NO conversion and (b) DeNOx index of the materials.

The conversion rates of NO to NO_2_ and by-products have been calculated (Figure 3a). The conversion rates of NO to NO_2_ of g-C_3_N_4_, MgO, 1% MgO@g-C_3_N_4_, 3% MgO@g-C_3_N_4_, and 5% MgO@g-C_3_N_4_ are 34.6%, 9.6%, 34.9%, 21.9%, and 25.8%, respectively. Besides, the rates of converting NO to by-products of g-C_3_N_4_, MgO, 1% MgO@g-C_3_N_4_, 3% MgO@g-C_3_N_4_, and 5% MgO@g-C_3_N_4_ are 28.2%, 7.3%, 34.4%, 53.5%, and 46.3%, respectively. In this study, the by-products are defined as any nitrogen species (e.g., N_2_O_5_, N_2_O, and NO_3_^−^) except NO_2_, which are unstable and can be absorbed by plants [46]. MgO generates the lowest amount of NO_2_ and by-products due to the lowest photocatalytic NO removal efficiency (16.8%). 3% MgO@g-C_3_N_4_ has the lowest NO_2_ (21.9%) and highest by-product (53.5%) generation. In addition, the NO_2_ generation of g-C_3_N_4_ is almost equal to that of 1% MgO@g-C_3_N_4_. This can be explained by the low amount of MgO (only 1%). 3% MgO@g-C_3_N_4_ shows the highest photocatalytic NO removal efficiency with the lowest NO_2_ generation, which indicates a possible future application of 3% MgO@g-C_3_N_4_. Also, the DeNOx index values have been calculated according to Equation 4 [47]. The values of g-C_3_N_4_, MgO, 1% MgO@g-C_3_N_4_, 3% MgO@g-C_3_N_4_, and 5% MgO@g-C_3_N_4_ are −201.6%, −58.2%, −160.8%, 47.4%, and −25.8%, respectively.

### XRD and FTIR analyses

**Figure 4:**
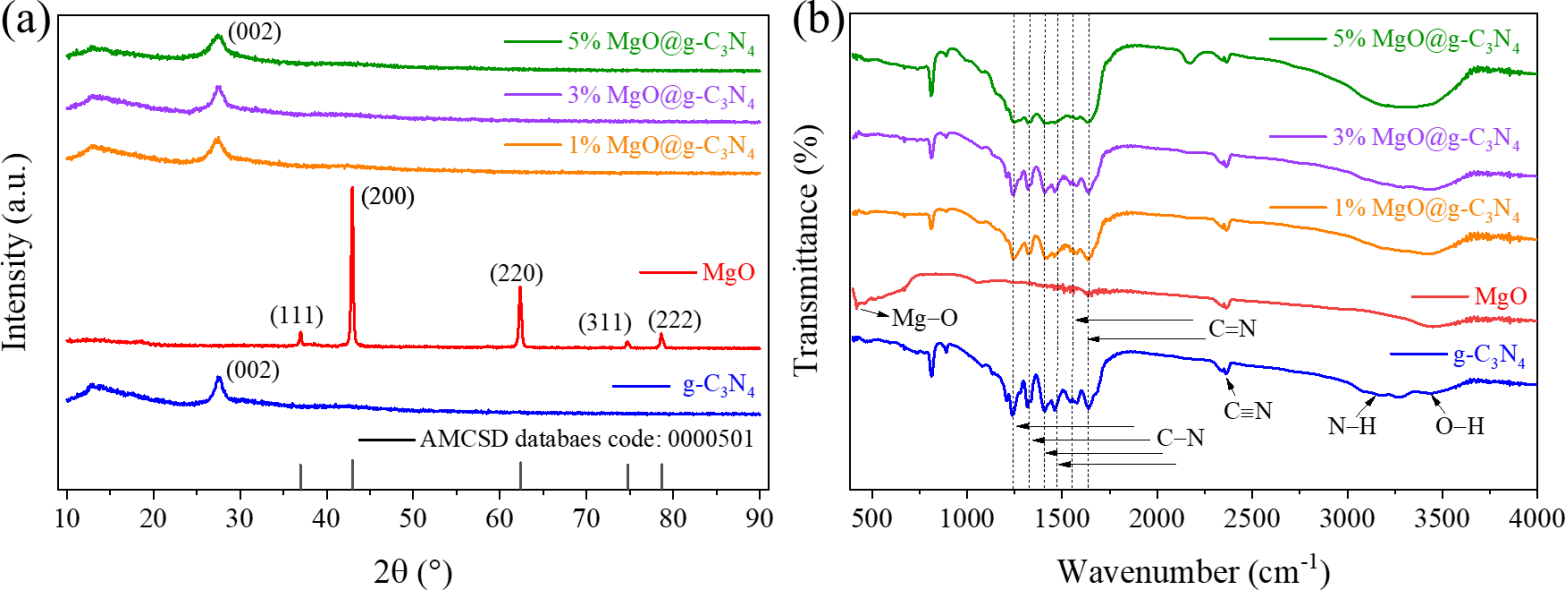
(a) XRD patterns and (b) FTIR spectra of the materials.

XRD patterns of the synthesized materials are shown in Figure 4a. There are two distinct diffraction peaks at 2θ = 13° and 27.4°, which were assigned to the (100) and (002) planes of g-C_3_N_4_, respectively [48]. Diffraction peaks of the pure MgO sample are detected at 36.9°, 42.9°, 62.5°, 74.8°, and 78.7°, which were attributed to the (111), (200), (220), (311), and (222) planes, respectively [AMCS: 000501] [49–50]. All MgO@g-C_3_N_4_ samples show the characteristic peaks of g-C_3_N_4_. No impurities are detected in the MgO@g-C_3_N_4_ samples. There are no reflections of MgO in all MgO@g-C_3_N_4_ samples, due to the low amount of MgO in the MgO@g-C_3_N_4_ samples. This agrees with previous studies, in which characteristic peaks of MgO were only detected when MgO amounts higher than 5% had been added [33,51].

Figure 4b shows the FTIR spectra of g-C_3_N_4_, MgO, and MgO@g-C_3_N_4_. For pure g-C_3_N_4_, the broad peak in the range of 3000–3600 cm^−1^ was attributed to the stretching vibrations of N–H and O–H bonds, indicating the existence of amino groups and adsorbed water molecules in the material [32,52]. The characteristic peaks at 1240, 1320, 1407, and 1465 cm^−1^ were associated with the stretching vibrations of aromatic C–N bonds, and the typical peaks at 1562 and 1642 cm^−1^ characterize the presence of C=O bonds [53–54]. In addition, the characteristic peak at 810 cm^−1^ matches with the typical breathing mode of triazine. After adding MgO, the distinct peaks of all MgO@g-C_3_N_4_ heterojunctions are similar to that of pure g-C_3_N_4_, indicating that the crystal structure of g-C_3_N_4_ remains unchanged. In addition, the small peak at 419 cm^−1^ proves the presence of MgO in MgO@g-C_3_N_4_ [55].

### SEM and TEM analyses

**Figure 5:**
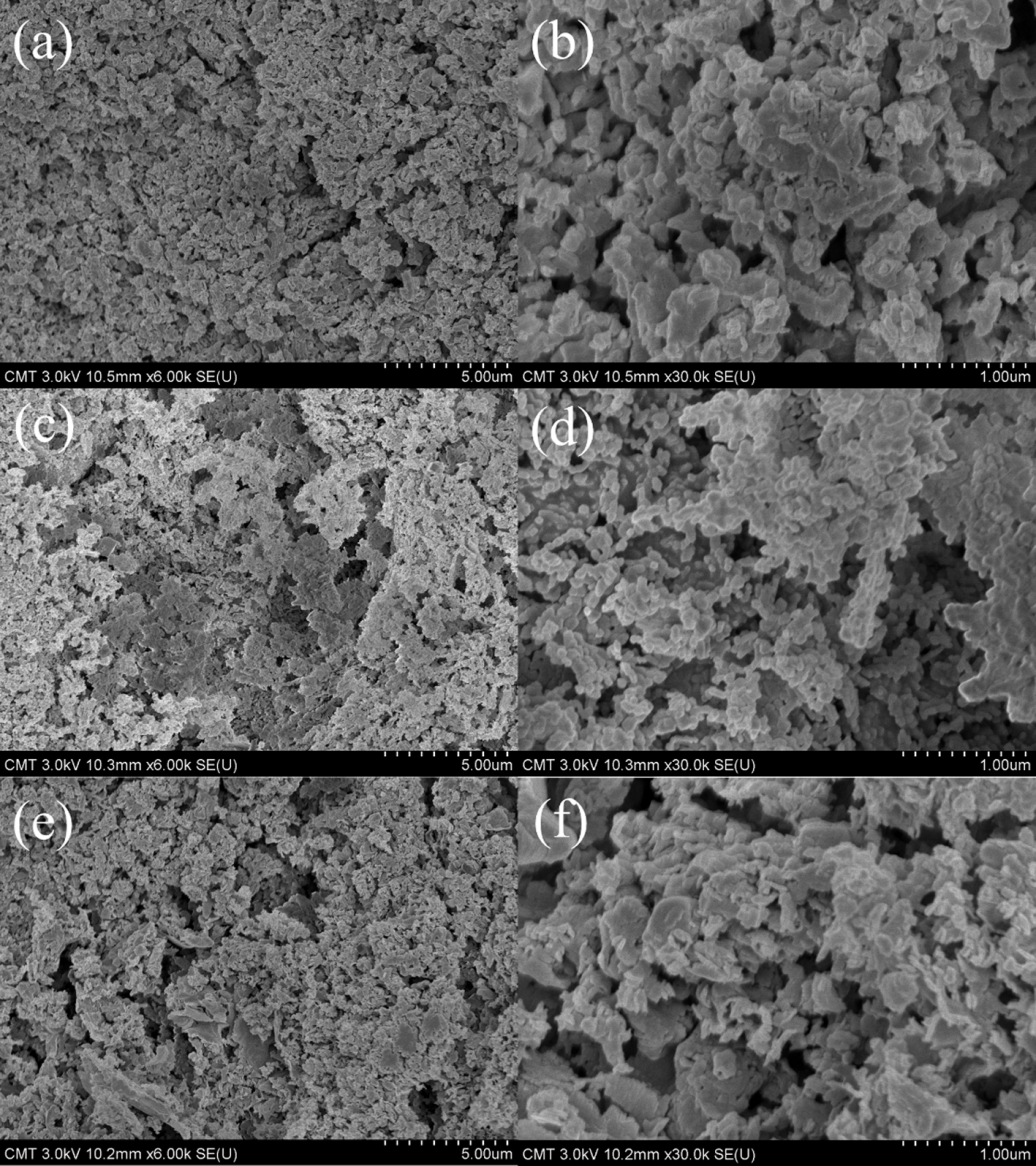
SEM images of (a, b) 3%MgO@g-C_3_N_4_, (c, d) MgO, and (e, f) g-C_3_N_4_.

**Figure 6:**
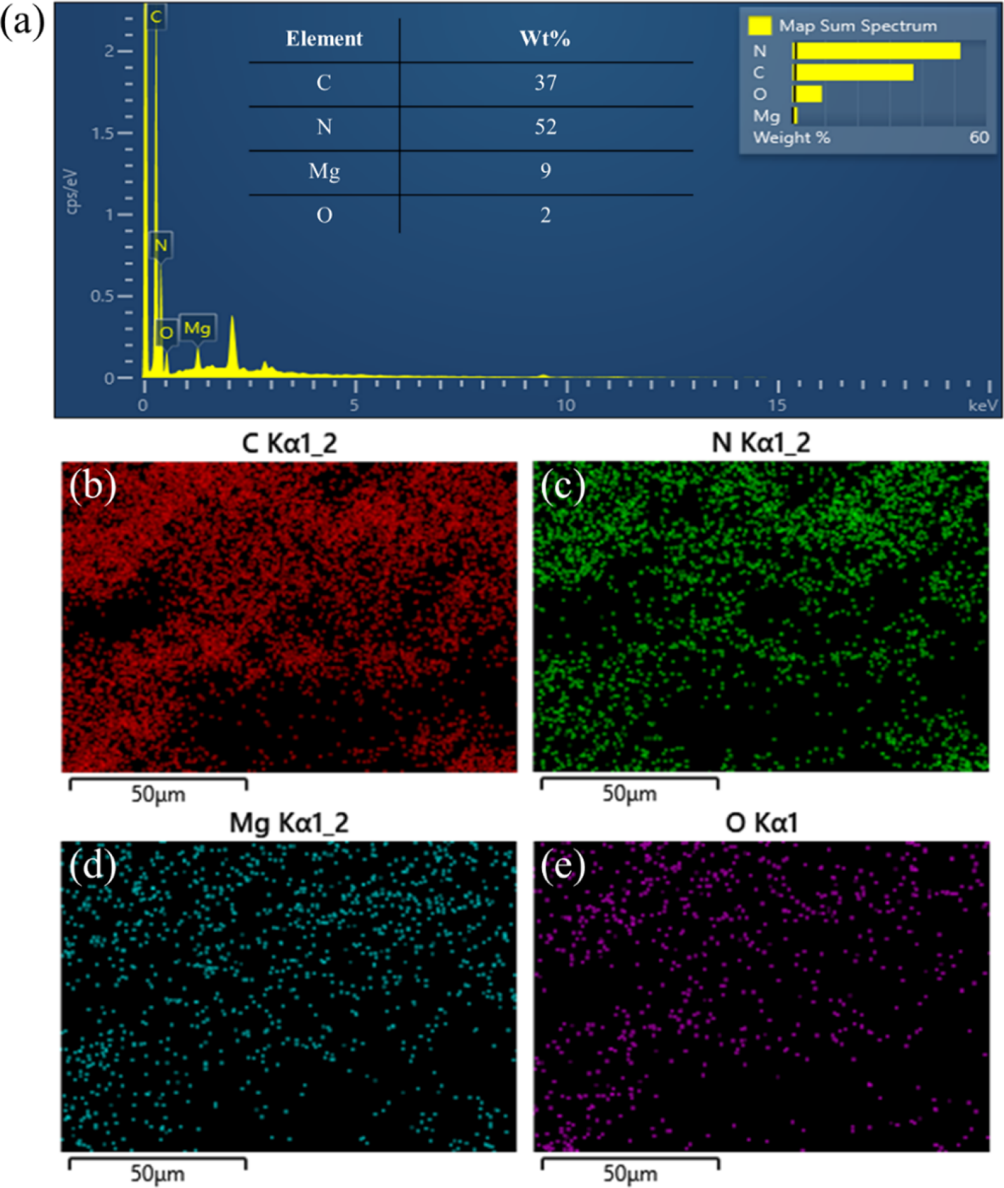
(a) Elemental composition and (b–e) EDS mappings of 3% MgO@g-C_3_N_4_.

**Figure 7:**
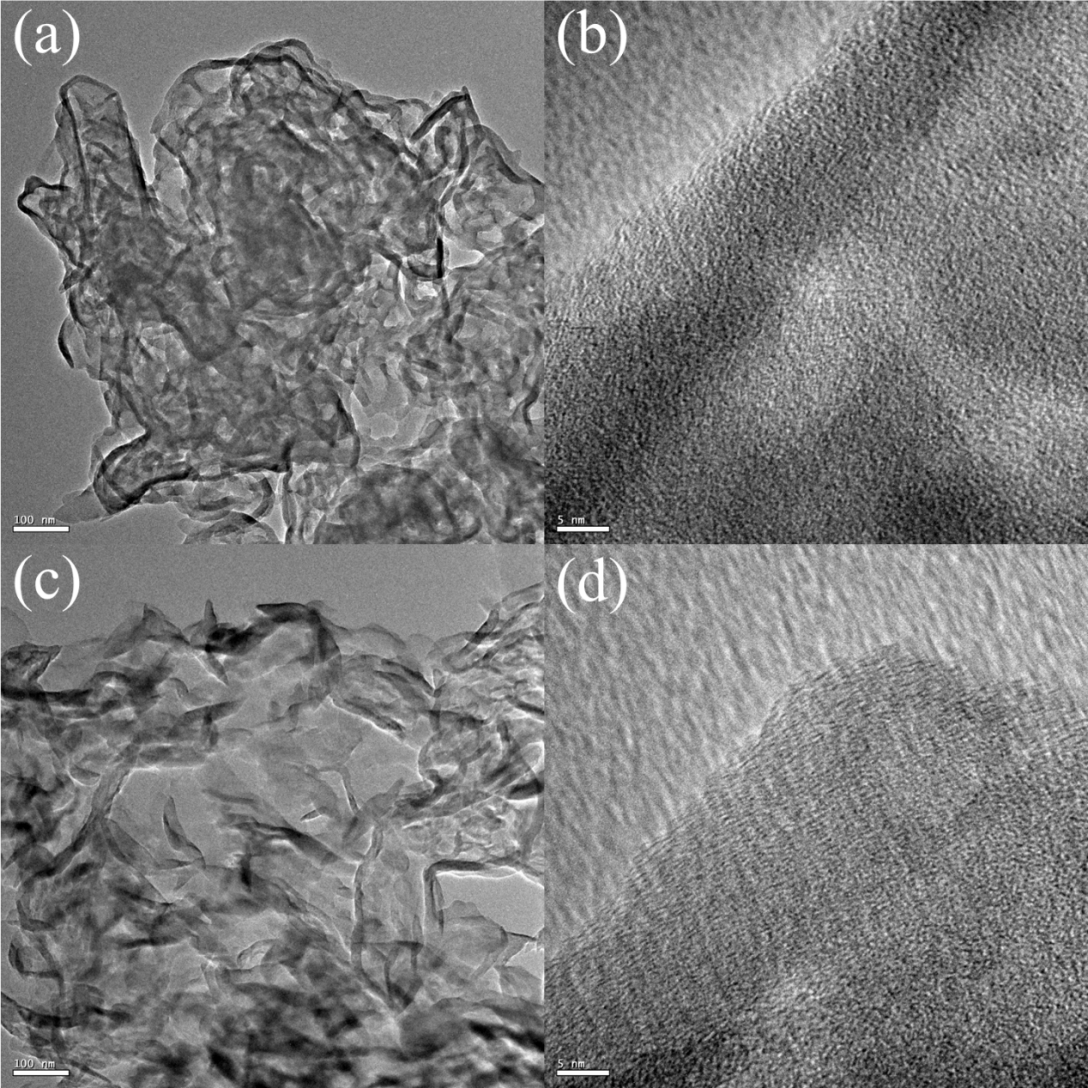
TEM and HR−TEM images of (a, b) MgO@g-C_3_N_4_ and (c, d) g-C_3_N_4_.

The morphology of g-C_3_N_4_, MgO, and 3%MgO@g-C_3_N_4_ has been determined through SEM and TEM analyses. The typical bulk structure of g-C_3_N_4_ is shown in Figure 5e,f. The difference between the morphologies of MgO and g-C_3_N_4_ is difficult to observe by SEM (Figure 5c,d). Figure 5a and Figure 5b show that the morphology of 3%MgO@ g-C_3_N_4_ is similar to that of pure g-C_3_N_4_. The results indicate that the morphology of 3% MgO@g-C_3_N_4_ is identical to that of g-C_3_N_4_ because the added amount of MgO is very low, as determined by EDS mapping. The EDS mapping images of 3% MgO@g-C_3_N_4_ are shown in Figure 6 and Figure S2 (Supporting Information File 1). The weight percentages of C, N, Mg, and O are 37, 52, 9, and 2 wt %, respectively. The weight fractions of Mg and O are the lowest, indicating that the amount of MgO in the MgO@g-C_3_N_4_ sample is too low. The shape of g-C_3_N_4_ is easy to observe in Figure 7c,d. However, the shape of MgO is complicated to determine by TEM and HR-TEM (Figure 7a,b). These results prove the presence of MgO in the compound.

### Chemical state analysis

**Figure 8:**
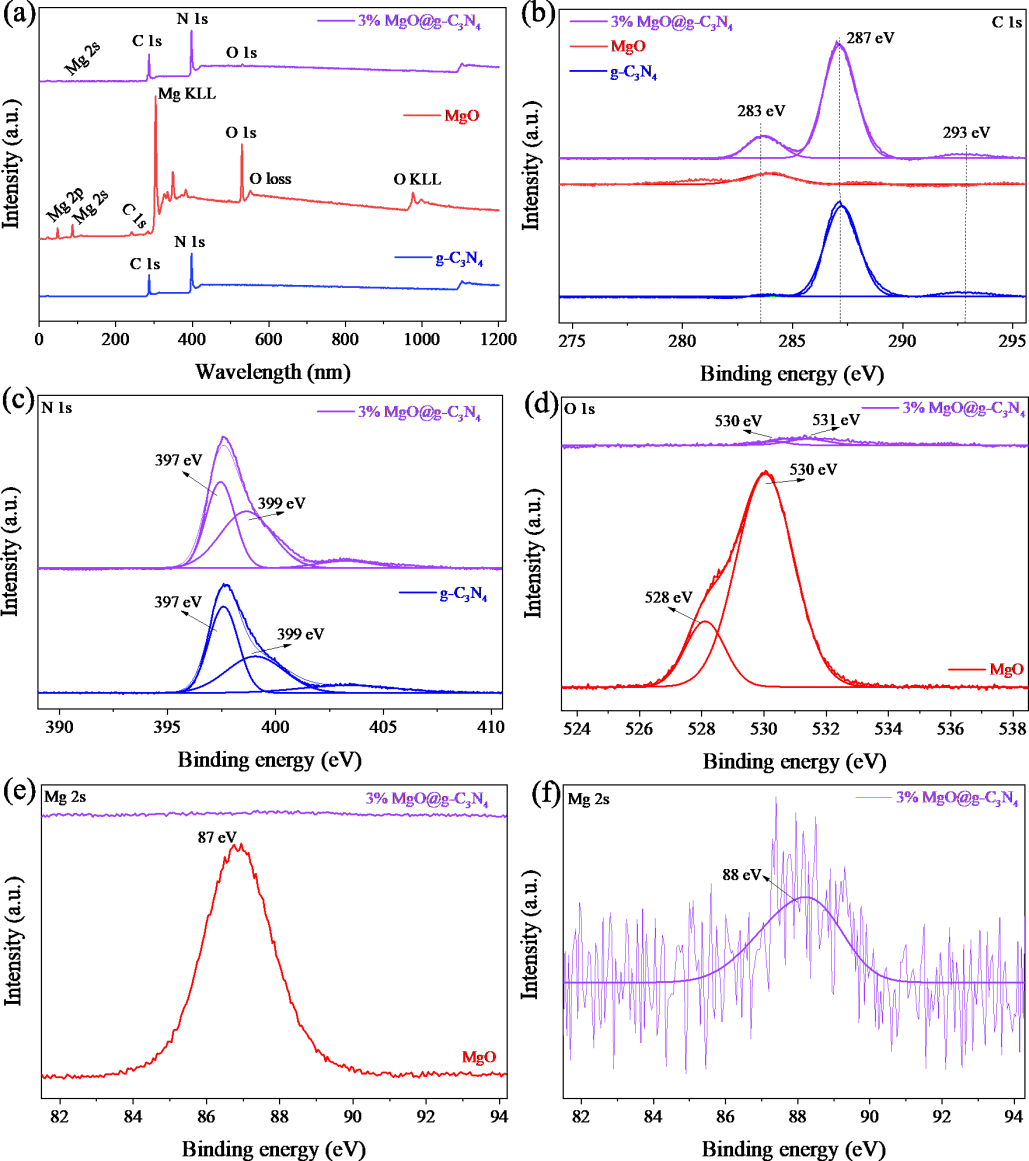
The XPS survey (a), HR−XPS C1s (b), N 1s (c), O 1s (d), C 1s (c), and Mg 2s (e, f) of the materials.

XPS and HR-XPS have been employed to determine the chemical states of the materials. XPS survey scans of g-C_3_N_4_ MgO and 3% MgO@ g-C_3_N_4_ are shown in Figure 8a. The peaks at 87 and 530 eV were assigned to the Mg 2s and O 1s levels of MgO, respectively. The peaks at 287 and 397 eV were assigned to the C 1s and N 1s levels of g-C_3_N_4_, respectively [12,56–57]. In the MgO sample, the peaks of Mg 2p, Mg KLL, O loss, and O KLL levels are observed at 46, 304, 555, and 978 eV, respectively [58–59]. The HR-XPS of the C 1s level are shown in Figure 8b. The peak at 283 eV was assigned to the C–C coordination in MgO, and the peak at 287 eV was assigned to N–C=N bonds of g-C_3_N_4_. The latter peak only appears in g-C_3_N_4_ and 3% MgO@g-C_3_N_4_. However, the former peak of MgO and g-C_3_N_4_ occurs only in 3% MgO@g-C_3_N_4_. The C 1s peaks of the materials do not change during the pyrolysis. The HR-XPS of the N 1s level of the materials is shown in Figure 8c. The peaks at 397 and 399 eV correspond to the C–N=C bonds and the N–(C)_3_ structures of g-C_3_N_4_, respectively [60]. Figure 8d shows the peak of Mg–O bonds in MgO at 398 eV [61]. The Mg 2s peaks of MgO are shown in Figure 8e and Figure 8f. The peaks at 87 eV (Figure 8e) and 88 eV (Figure 8f) confirm the metallic state of Mg [62]. The peaks of the O 1s and Mg 2s levels in 3% MgO@g-C_3_N_4_ are too weak.

### Optical properties

**Figure 9:**
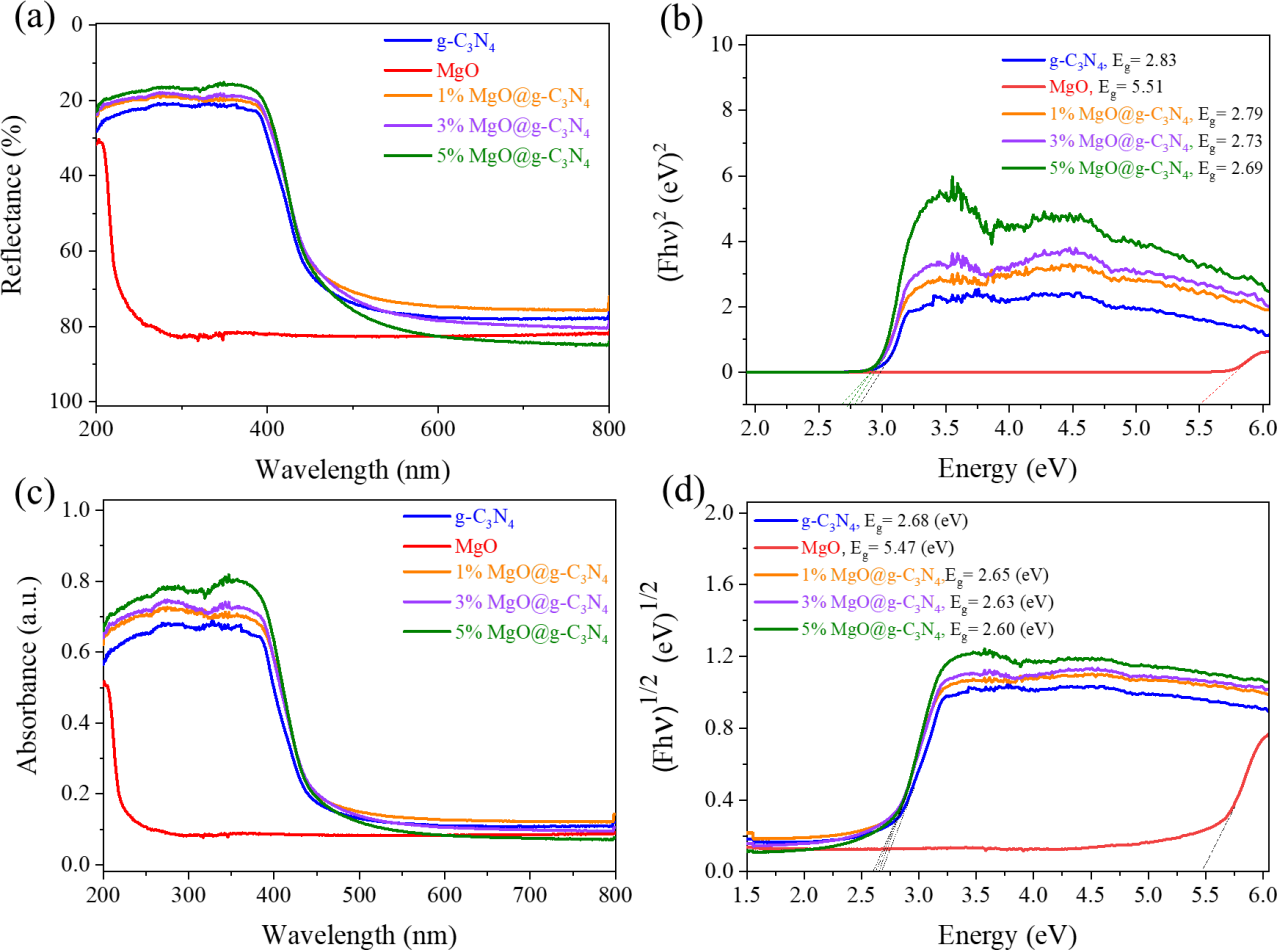
(a) DRS reflectance spectra, (b) direct bandgap, (c) DRS absorbance spectra, and (d) indirect bandgap of the materials.

DRS spectra have been measured to understand the optical absorption characteristics of the materials (Figure 9). The g-C_3_N_4_ sample significantly absorbs light at a peak around 440 nm, corresponding to the direct and the indirect bandgap of 2.83 and 2.68 eV, respectively (Figure 9b,d). MgO with a wide bandgap shows absorption below 400 nm in both Kubelka–Munk and Tauc plots (Figure 9a,c). After adding MgO to g-C_3_N_4_, the absorption of the MgO@ g-C_3_N_4_ samples slightly shifts to the visible light region. The optical direct and indirect bandgap energy is slightly reduced, corresponding to the increase in MgO fraction. The indirect bandgap energies of 1% MgO@g-C_3_N_4_, 3% MgO@g-C_3_N_4_, and 5% MgO@g-C_3_N_4_ are 2.79, 2.73, and 2.69 eV, respectively. The trend of the direct bandgap is the same. The bandgap of the materials reduces with increasing amounts of added MgO. The bandgap reduction can be attributed to Mg−N bonds in the MgO@g-C_3_N_4_ materials, which promote charge transport, thus, increasing the photocatalytic efficiency [33,51]. Generally, smaller bandgaps lead to better light absorption, indicating the high photocatalytic activity of MgO@g-C_3_N_4_ under visible light and also the existence of MgO in the MgO@g-C_3_N_4_ heterojunction materials. However, with a smaller bandgap, the recombination of e^−^–h^+^ pairs will be faster, which decreases the photocatalytic activity of the materials [63]. As shown in Figure 9a and Figure 9c, the materials mostly absorb in the UV range (200–400 nm), with a sudden decrease in the visible range. The absorbance of 3% MgO@g-C_3_N_4_ is more substantial than the absorbance of 1% MgO@g-C_3_N_4_ and 5% MgO@g-C_3_N_4_ in the UV and visible ranges. These results indicate that the higher photocatalytic NO removal efficiency of the 3% MgO@g-C_3_N_4_ strongly depends on the optical properties.

### Photoluminescence

**Figure 10:**
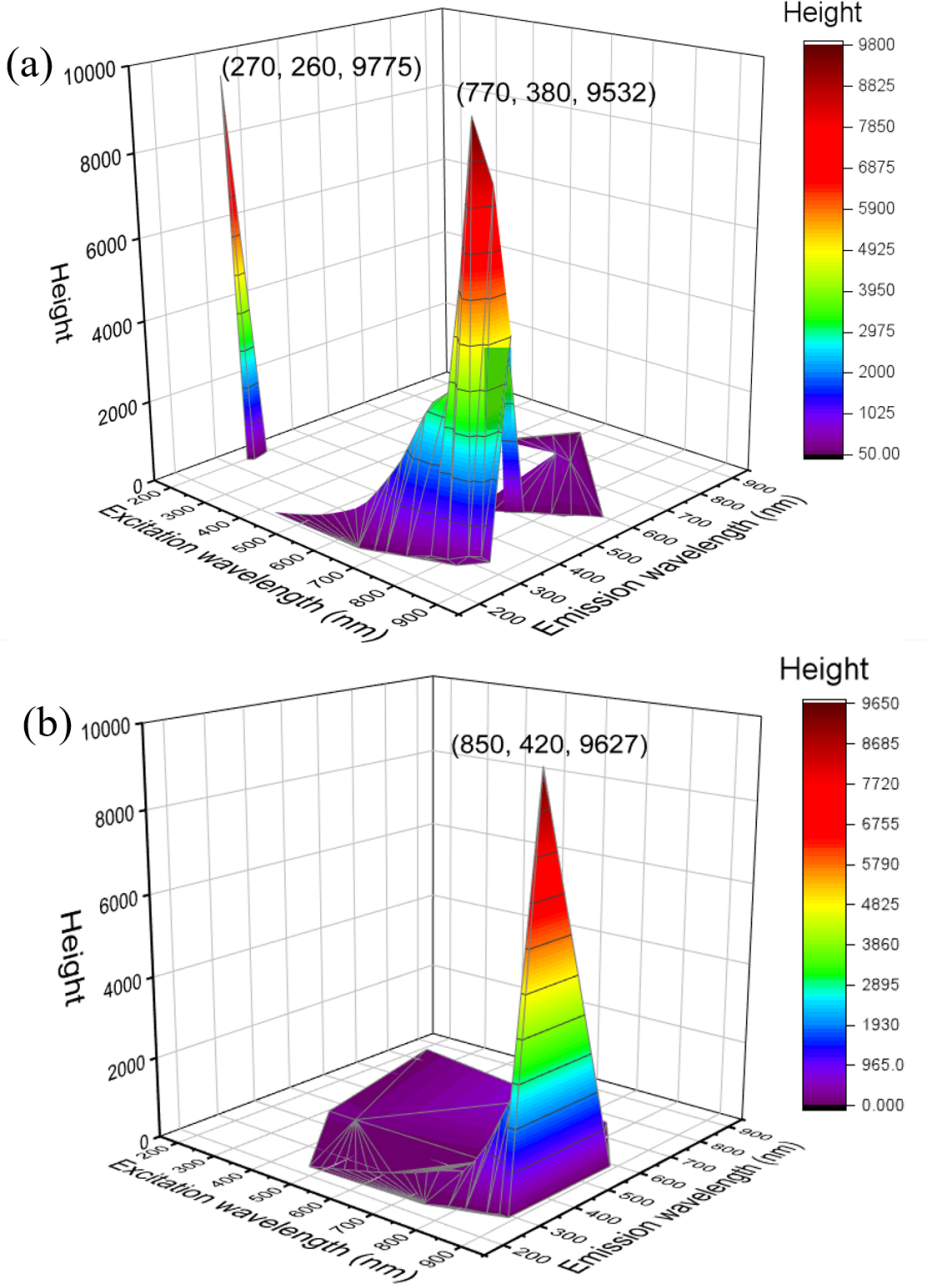
3D fluorescence scan of (a) MgO and (b) 3% MgO@g-C_3_N_4_.

Fluorescence spectra of MgO and 3% MgO@g-C_3_N_4_ are shown in Figure 10a and Figure 10b, respectively. MgO shows strong fluorescence at 270 nm with an excitation wavelength (270 nm) in the UV range. MgO also shows another emission wavelength at 380 nm with an excitation wavelength (770 nm) in the visible range, which could be caused by the native structural defects in MgO [18]. 3% MgO@g-C_3_N_4_ shows intense fluorescence at 420 nm via excitation at 850 nm, due to the recombination of charge carriers. The photogenerated electrons from the valance band (VB) of g-C_3_N_4_ migrate to the conduction band (CB). The excited electrons in the CB of g-C_3_N_4_ can then return to energy bands between the CB and VB of g-C_3_N_4_ to produce an emission with an energy of about 1.5 eV. The energy band in g-C_3_N_4_ can be attributed to transitions between C atoms and N atoms [64–66]. Also, Liang and co-workers reported that the recombination of the e^−^–h^+^ pairs could be inhibited by doping MgO into g-C_3_N_4_ [32]. When MgO is added, the defect concentration increases and Mg and O vacancies are generated in MgO@g-C_3_N_4_. These defects work as the electron traps, which enhance the capacity to separate photogenerated e^−^−h pairs in MgO@g-C_3_N_4_. The photogenerated e^−^–h^+^ pairs in the defects also contribute to the photocatalytic reaction [67–68].

### Photocatalytic mechanism

**Figure 11:**
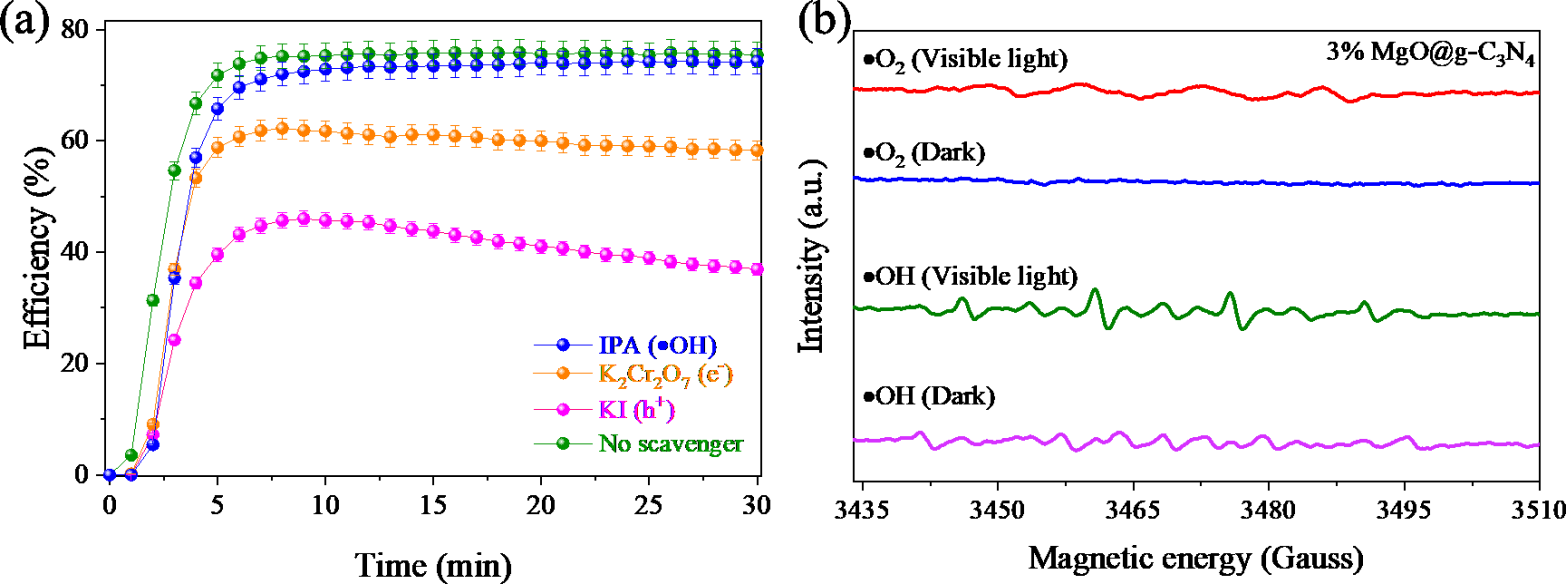
(a) Trapping test results of the materials and (b) detection of radicals over over 3% MgO@g-C_3_N_4_ by ESR.

Trapping experiments were carried out to evaluate the involvement of electrons, holes, and reactive oxygen species. The used trapping agents were KI (h^+^), K_2_Cr_2_O_7_ (e^−^), and IPA (•OH). Figure 11a shows the reduction in efficiency when different scavengers are present. The photocatalytic NO degradation efficiency decreases significantly from 75.4% to 36.4% in the presence of KI. K_2_Cr_2_O_7_ as electron scavenger also reduces the NO decomposition by about 1.3 times. The weak contribution of •OH radicals in the NO degradation is clearly shown, with a reduction in efficiency by only about 1%. Hence, electrons and holes are the main contributors to the photocatalytic NO degradation.

Also, ESR was used to determine accurately the reaction mechanism of the material. Figure 11b shows that under visible light and using DMPO–H_2_O and DMPO–OH, the material generates •OH and •O_2_ radicals. In contrast, only •OH radicals are generated in the dark, but to a very low extent. Hence, the generation of •O_2_ radicals contributes significantly to the photocatalytic NO degradation efficiency.

A photocatalysis mechanism of MgO@g-C_3_N_4_ is proposed taking into account the results of the DRS and ESR analyses and trapping tests. Because of the large bandgap of MgO, only g-C_3_N_4_ generates e^−^–h^+^ pairs under visible light (Equation 8). The holes can degrade NO directly by oxidizing NO into NO_2_ (Equations 9 and 10) [47,69]. Simultaneously, at the CB of g-C_3_N_4_, electrons are excited and react with O_2_ to produce •O_2_^−^ radicals. In addition, these electrons also migrate across the CB of MgO, creating excess electrons in MgO and avoiding the recombination at g-C_3_N_4_. Then, the photogenerated electrons react with O_2_ to produce •O_2_^−^ radicals (Equation 9). These •O_2_^−^ radicals decompose NO to NO_3_ and prevent the formation of NO_2_ (Equation 10). Also, •O_2_^−^ reacts with H_2_O to produce •HO_2_ radicals and OH^−^ (Equation 11). Then, the •HO_2_ decomposes NO to form NO_2_ and •OH (Equation 12). Also, •HO_2_ also reacts with H_2_O and electrons to produce H_2_O_2_ (Equation 13). Finally, H_2_O_2_ generates two •OH radicals to decompose NO (Equations 16–19) [70–71].


[8] $${\text{MgO@g-C}}_{3}{\text{N}}_{4}+h\nu \to {\text{MgO@g-C}}_{3}{\text{N}}_{4}\left({\text{e}}^{-}{}_{\text{(CB)}}+{\text{h}}^{+}{}_{\text{(VB)}}\right),$$

[14] $${\text{h}}^{+}{}_{\text{(VB)}}+\text{NO}+{\text{H}}_{2}\text{O}\to {\text{HNO}}_{2}+{\text{H}}^{+},$$

[15] $$2{\text{HNO}}_{2}\to {\text{NO}}_{2}+\text{NO}+{\text{H}}_{2}\text{O},$$

[9] $${\text{e}}^{-}{}_{\text{(CB)}}+{\text{O}}_{2}\to \cdot {\text{O}}_{2}{}^{-},$$

[10] $$\cdot {\text{O}}_{2}{}^{-}+\text{NO}\to {\text{NO}}_{3}{}^{-},$$

[11] $$\cdot {\text{O}}_{2}{}^{-}+{\text{H}}_{2}\text{O}\to \cdot {\text{HO}}_{2}{\text{+OH}}^{-},$$

[12] $$\text{NO}+\;\cdot {\text{HO}}_{2}\to {\text{NO}}_{2}+\;\cdot \text{OH},$$

[13] $$\cdot {\text{HO}}_{2}+{\text{H}}_{2}\text{O}+{\text{e}}^{-}{}_{\text{(CB)}}\to {\text{H}}_{2}{\text{O}}_{2}+{\text{OH}}^{-},$$

[16] $${\text{H}}_{2}{\text{O}}_{2}\to 2\cdot \text{OH},$$

[17] $$\text{NO}+\;\cdot \text{OH}\to {\text{HNO}}_{2},$$

[18] $${\text{HNO}}_{2}+\;\cdot \text{OH}\to {\text{NO}}_{2}+{\text{H}}_{2}\text{O},$$

[19] $${\text{NO}}_{2}+\;\cdot \text{OH}\to {\text{HNO}}_{3}.$$

## Conclusion

MgO@g-C_3_N_4_ heterojunction materials were effectively synthesized by one-step pyrolysis of commercial MgO and urea. The photocatalytic efficiencies of the synthesized materials were increased dramatically by mixing MgO and g-C_3_N_4_. 3% MgO@g-C_3_N_4_ possessed the highest photocatalytic NO removal efficiency, reaching 75.4%. In addition, the photocatalytic NO removal efficiency of the MgO@g-C_3_N_4_ heterojunction materials was decreased when the amount of MgO exceeded 3%. An enhanced apparent quantum efficiency of (6.7 × 10^−4^)% as well as an extended lifetime of photogenerated electrons based on the heterojunction structure were obtained by combining MgO and g-C_3_N_4_. Moreover, the conversion rates of NO to NO_2_ and by-products of the 3% MgO@g-C_3_N_4_ were the lowest (21.9%) and the highest (53.5%), respectively. Also, 3% MgO@ g-C_3_N_4_ showed high reusability after a five-cycle recycling test, when the photocatalytic NO removal efficiency decreased only by 7.1%. The results indicated that 3% MgO@g-C_3_N_4_ could be applied in the future as an excellent photocatalyst with high removal efficiency and low generation of toxic products. FTIR, XPS, and EDS measurements were carried out to confirm the presence of MgO in the MgO@g-C_3_N_4_ heterojunctions. Although MgO was difficult to determine in the MgO@g-C_3_N_4_ heterojunctions, the addition of MgO affected the optical properties of the MgO@g-C_3_N_4_ heterojunctions. The DRS result showed that the bandgap of the MgO@g-C_3_N_4_ heterojunctions decreased by adding larger amounts of MgO. The light absorption of 3% MgO@g-C_3_N_4_ was higher than that of 1% MgO@g-C_3_N_4_ and 5% MgO@g-C_3_N_4_ in the visible and UV range, which increased the photocatalytic performance under visible light irradiation. The PL results confirmed the presence of vacancies in the MgO@g-C_3_N_4_ heterojunctions. MgO@g-C_3_N_4_ is promising for large-scale fabrication via this simple and fast method. This study provides a new way to synthesize a MgO@g-C_3_N_4_ heterojunction materials and an understanding of the photocatalytic mechanism of the MgO@g-C_3_N_4_ heterojunction applied in the removal of NO.

## Supporting Information

File 1 Additional figures.
