# Supplementary material for: Rapid fabrication of MgO@g-C3N4 heterojunctions for photocatalytic nitric oxide removal
Source: Beilstein J Nanotechnol. 2022 Oct 18;13:1141–54. doi: 10.3762/bjnano.13.96 (PMC9592965; doi:10.3762/bjnano.13.96)
Supplement: File 1 — Additional figures. [file Beilstein_J_Nanotechnol-13-1141-s001.pdf]

BEILSTEIN JOURNAL OF NANOTECHNOLOGY

Supporting Information

for

Rapid fabrication of MgO@g-C_3N_4 heterojunctions for photocatalytic nitric oxide removal

Minh-Thuan Pham, Duyen P. H. Tran, Xuan-Thanh Bui and Sheng-Jie You

Beilstein J. Nanotechnol. **2022,** *13,* 1141–1154. doi:10.3762/bjnano.13.96

Additional figures

License and Terms: This is a supporting information file under the terms of the Creative Commons Attribution License (https://creativecommons.org/licenses/by/4.0). Please note that the reuse, redistribution and reproduction in particular requires that the author(s) and source are credited and that individual graphics may be subject to special legal provisions.
The license is subject to the *Beilstein Journal of Nanotechnology* terms and conditions: (https://www.beilstein-journals.org/bjnano/terms)

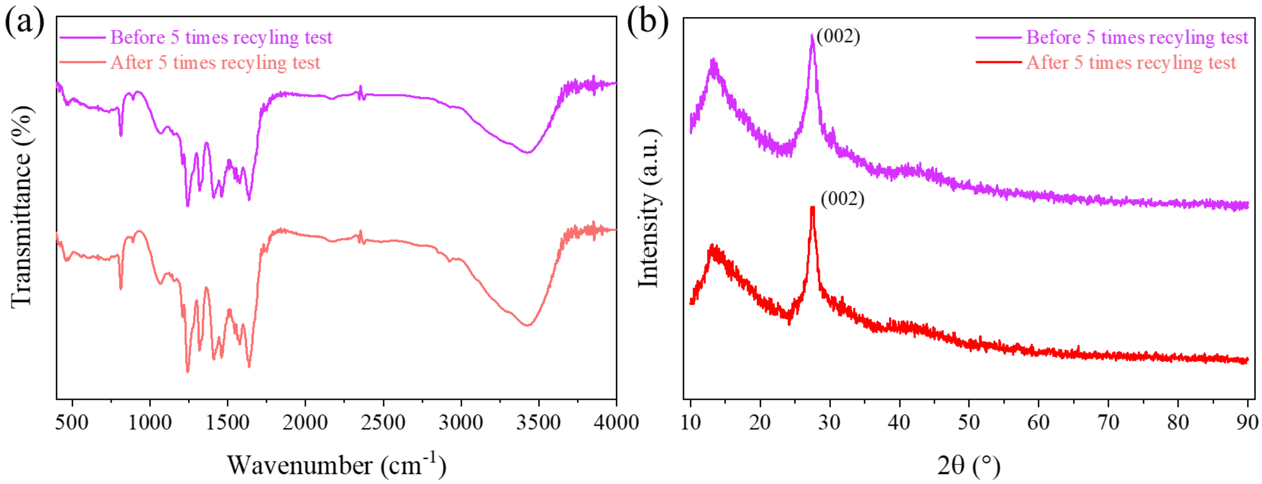

Figure S1: (a) FTIR spectra and (b) XRD patterns of 3%MgO@g-C_3N_4 before and after the recycling tests.

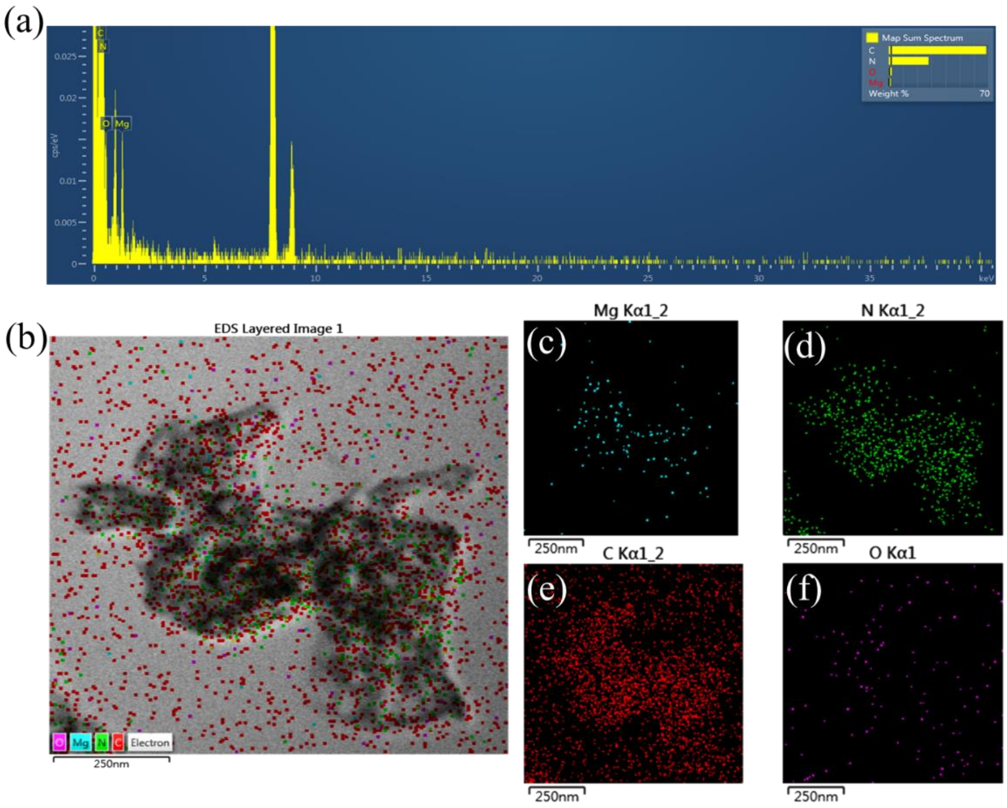

Figure S2: TEM EDS mapping of 3%MgO@g-C_3N_4.
